# School‐based interventions on Mpox: A scoping review

**DOI:** 10.1002/hsr2.1334

**Published:** 2023-06-12

**Authors:** Jimoh Amzat, Kehinde K. Kanmodi, Kafayat Aminu, Eyinade A. Egbedina

**Affiliations:** ^1^ Department of Sociology Usmanu Danfodiyo University Sokoto Nigeria; ^2^ Department of Sociology University of Johannesburg Johannesburg South Africa; ^3^ Faculty of Dentistry University of Puthisastra Phnom Penh Cambodia; ^4^ Cephas Health Research Initiative Inc Ibadan Nigeria; ^5^ School of Health and Life Sciences Teesside University Middlesbrough UK; ^6^ Centre for Child and Adolescent Mental Health, University College Hospital Ibadan Nigeria

**Keywords:** child health, children, global health, intervention, mpox, school, scoping review

## Abstract

**Background and Aims:**

The 2022 multicountry mpox outbreak necessitated the declaration of mpox as a public health emergency. This is the first time a wide mpox spread and human‐to‐human transmission are recorded in several countries outside West and Central Africa. The outbreak reveals a strong need for wider intervention to increase awareness and control measures on mpox, especially in schools. This scoping review aims to summarize the existing evidence concerning school‐based interventions on mpox globally.

**Methods:**

The review methodology was based on the Arksey and O'Malley guidelines and it was reported in strict adherence with the PRISMA‐ScR checklist. Ten databases were searched to retrieve literature relevant to the review topic. Thereafter, the retrieved literature were deduplicated and screened for inclusion into the review based on a set of eligibility criteria. Only one journal paper, a short communication on the national monkeypox outbreak in England, satisfied the selection criteria and was included in the review. Data extracted from the included paper were collated, summarized, and presented.

**Results:**

The paper described how suspected cases of mpox infection were managed in some school settings through vaccination and self‐isolation approaches and revealed a low (11%) mpox vaccination uptake rate in school‐setting. The preventive methods adopted, such as the exclusion of exposed persons from school (in three school settings) and separation of those exposed those who were not in contact with the affected persons (one school setting), played a major role in the low transmission rate reported. This review also found a huge dearth of literature on school‐based interventions on mpox despite its global spread.

**Conclusion:**

With the call for a multisectoral approach in the fight against mpox, it pays to leverage on the potential of school settings in public health actions against mpox.

## 1 INTRODUCTION

The monkeypox (now known as mpox) virus, first identified in 1970, is an orthopoxvirus, which causes infections with symptoms similar to smallpox, although less severe.^1^ Mpox is traditionally found in countries of central and west Africa with confirmed cases in the Central African Republic, Democratic Republic of the Congo, Liberia, Nigeria, Republic of the Congo, and Sierra Leone.^2^ Unfortunately, most of the mpox endemic countries do not have adequate control infrastructure for prevention, detection and prevention.^3^ Mpox is mostly transmitted from animals (such as squirrels, Gambian pouched rats, dormice, different species of monkeys and others) to humans. Human‐to‐human transmission occurs through contact with body fluids (including sexual contacts), lesions on the skin or internal mucosal surfaces (in the mouth or throat), respiratory droplets and contaminated objects.^4^, ^5^

Globalization, a major driver of human mobility, has opened up the world, which incidentally facilitates the transmission of infectious diseases.^6^ Most infectious diseases constitute significant and acute global health challenges. Predictably, the multicountry outbreak (including Europe and North America) of mpox necessitated the declaration of mpox as a public health emergency.^5^ As of the end of January 2023, the 2022 outbreak has resulted in 85,499 confirmed cases in 110 countries with 89 deaths.^7^ The spread has cut across the globe with the 10 most affected countries, accounting for over 85% being outside Africa including the United States of America with 29,891 cases, Brazil with 10,739 cases;, Spain with 7518 cases, France with 4128 cases, Colombia with 4066 cases, Mexico with 768 cases, the United Kingdom with 3735 cases, Peru with 3723 cases, Germany with 3692 cases, and Canada with 1460 cases.^7^ Venkatesan^5^ observed that the 2022 mpox outbreak has the highest recorded incidence compared to previous outbreaks. It is also the first time there is high incidence outside the West and Central Africa. The data shows a strong need for wider intervention to increase awareness and induce appropriate responses from the global population.

Globally, there is a low prevalence of mpox among school‐aged children^8^, ^9^ but intense and possibly prolonged physical contact among the school‐aged in schools and during activities such as sports and plays constitute a significant concern for any infectious diseases including mpox and COVID‐19 among others. Sam‐Agudu et al.^9^ reported an mpox epidemiological transition, initially with high prevalence among children (under 10 years old) but now more prevalent among adults and sexual minorities. The global prevalence among children is less than 2% but up to 40% among children in Africa.^7^ Children and adults also account for the highest mortality rates in Africa.^9^

While there is a strong need for multisectoral intervention, one significant way of reaching a wider population is a school‐based approach.^10^, ^11^ A school‐based intervention depicts measures implemented in classrooms or in schools to improve health and wellbeing and reducing risk or problem behavior of the students.^12^ School‐based intervention is a significant way of reaching school‐aged children which could be an indirect way of reaching their parents and other household members.^13^ Such an approach will enhance information spread and a form of engagement, with a significant effect on preventive and control actions.^14^ The school‐based approach is also pertinent because of the sexual dimension in mpox transmission.

The school setting has always been a significant space for health promotion to curtail the prevalence of both chronic and infectious diseases. The ultimate goal of health intervention in the school setting is to improve knowledge of risk factors, promote appropriate health behavior through personal interiorization of health knowledge and critical thinking about harmful effects of common risky behaviors.^15^, ^16^, ^17^ Pulimeno et al.^16^ observed that health promotion within the school setting could reduce the prevalence of measurable unhealthy outcomes and improve academic achievements. It is always a case of primary prevention and health promotion targeting early life and the school is an ideal setting of action.^16^ It is pertinent that several studies, including scoping reviews, have been conducted on mpox^18^, ^19^, ^20^; however, no known scoping review has been conducted as of the first quarter of 2023 to summarize evidence on school‐based interventions on the disease. The need for such scoping review evidence is very crucial as such findings will set the pace for further research on mpox prevention and control, and it will also inform the public health planning, implementation, and evaluation of future school‐based interventions on mpox. Hence, this study aims to conduct a scoping review of evidence on school‐based interventions on mpox across the world.

## 2 METHODS

### 2.1 Title and protocol registration and deviations from the protocol

The title and protocol of this scoping review was initially registered as systematic review with PROSPERO (CRD42023409593). However, we deviated from the initial protocol and changed the study design to a scoping review due to the appropriateness of a scoping review for this study.^21^

### 2.2 Review design

The rationale of this scoping review was to summarize the existing research evidence on school‐based interventions on Mpox.^21^ This review's methodology was based on the Arksey and O'Malley's^22^ guidelines for scoping reviews, and it was reported in accordance with the Preferred Reporting Items for Systematic Reviews and Meta‐Analyses extension for Scoping Reviews checklist.^23^ Additionally, the quality of this review's methodological process was ensured through strict adherence to the guidelines prescribed in the AMSTAR‐2 checklist.^24^

### 2.3 Identification of review question

This scoping review seeks to answer this principal question: What are the existing evidence on school‐based interventions on mpox?

### 2.4 Identification of literature

#### 2.4.1 Selection criteria

Below are the criteria for the inclusion and exclusion of literature in this scoping review:

##### Inclusion criteria

The criteria for the inclusion of literature into this scoping review are the following:

1. Peer‐reviewed journal publications on school‐based interventions on mpox
2. Journal publications of any type (letters, short communications, short reports, full‐length articles, etc.).
3. Journal publications with accessible full text.
4. Studies conducted in any part of the world.

##### Exclusion criteria

The criteria for the exclusion of literature into this scoping review are the following:

1. Publications that are not in peer‐reviewed journals (e.g., books, book chapters, media news reports, etc.).
2. Publications on school‐based interventions on Ebola, COVID‐19, and other public health problems.
3. Publications on mpox that are interventions that were not delivered in school settings.
4. Journal publications with inaccessible full text.

#### 2.4.2 Literature search strategy

On March 21, 2023, 10 electronic databases were systematically searched by two reviewers for literature on school‐based interventions on mpox; these databases include Child Development and Adolescent Studies (via EBSCO interface), CINAHL Ultimate (via EBSCO interface), APA PsycINFO (via EBSCO interface), APA PsycArticles (via EBSCO interface), Psychology and Behavioral Sciences Collection (via EBSCO interface), Dentistry and Oral Sciences Source (via EBSCO interface), SPORTDiscus with Full Text (via EBSCO interface), AMED—The Allied and Complementary Medicine Database (via EBSCO interface), SCOPUS, and PubMed. Relevant search terms and synonyms obtained from the Medical Subject Heading (MeSH) dictionary and Thesaurus were used for the search strategy, with the aid of Boolean operators (‘AND’ and ‘OR’) and without year and language limiters. The search strings used for the search strategy are illustrated in the Appendix (Tables A1, A2 and A3).

#### 2.4.3 Deduplication of literature

All the literature obtained from the database search was imported into the Rayyan web application and deduplicated.^25^

#### 2.4.4 Screening and selection of literature

After deduplication, the residual literature were screened for the identification of those literatures meeting the scoping review's inclusion criteria. The screening process was two‐staged and done by two reviewers (K. K. K. and E. A. E.) with the aid of the Rayyan web application.^25^ The first stage involved screening titles and abstracts for relevance while the second stage involved the full‐text screening for final inclusion into the scoping review. With strict adherence to the scoping review's selection criteria, only those literature found eligible were included in the review.

### 2.5 Data charting, collation, and summarization

Relevant data, including citation data (authors' names and publication year), study design, study objectives, study's location, population under study, sample size, findings, and conclusions, were charted from the included article by two reviewers using a bespoke data extraction sheet. Thereafter, the extracted data were collated, summarized and presented as results.

## 3 RESULTS

**Figure 1:**
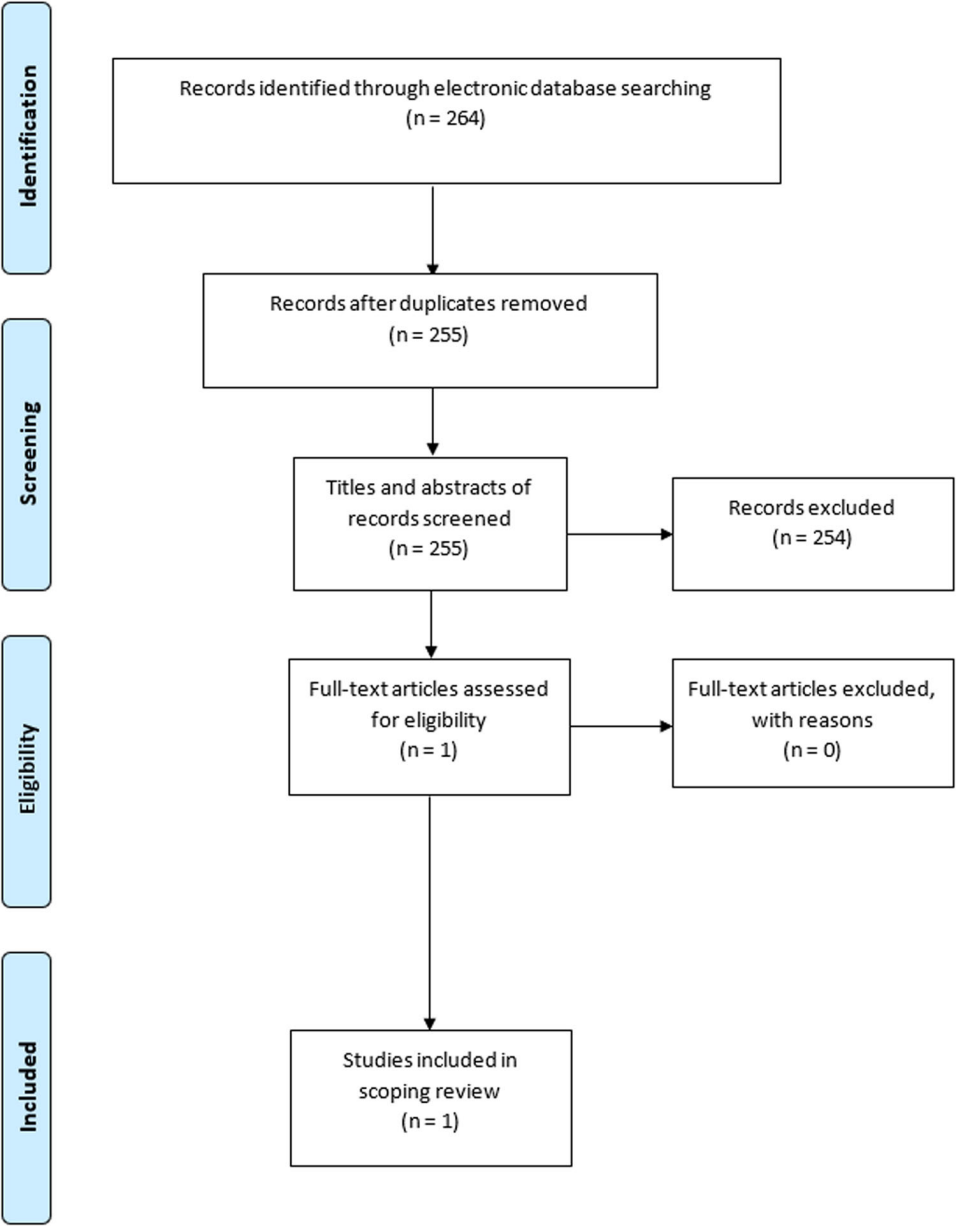
Flow chart diagram.

A total number of 264 publications were retrieved from the 10 databases, with the largest number of retrieved publications obtained from SCOPUS (*n* = 240) (Table 1). Nine duplicates were found and excluded, and the remaining 255 publications were subjected to screening. Of these 255 publications, only 1 publication was found eligible for inclusion in the scoping review (Figure 1).

**Table 1:** Total number of publications retrieved from each database search.

Database	Retrieved publications
SCOPUS	240
PubMed	14
APA PsycArticles (EBSCO interface)	0
APA PsycInfo (EBSCO interface)	3
AMED—The Allied and Complementary Medicine Database (EBSCO interface)	0
SPORTDiscus with Full Text (EBSCO interface)	1
Dentistry and Oral Sciences Source (EBSCO interface)	0
Child Development and Adolescent Studies (EBSCO interface)	3
CINAHL Ultimate (EBSCO interface)	3
Psychology and Behavioral Sciences (EBSCO interface)	0
Total: 10 Databases; 264 publications (including 9 duplicates)	

The only included literature is a rapid communication on national monkeypox virus (mpox virus) outbreak in England.^26^ The paper documented the exposure of some children and adults to infected persons in school settings between June and July 2022 and reported how suspected Mpox cases were managed. The review confirmed a low risk of mpox infection transmission in the student population. After the 28‐day follow‐up of suspected cases, no secondary mpox infection was recorded among the exposed school children (*n* = 380) and staff members (*n* = 100). This was attributed to their brief exposure and limited contact with the positive cases.

### 3.1 MPox prevention and control

The preventive method reported in the only included paper^26^ such as the exclusion of exposed persons from school (in three school settings) and separation of those exposed those who were not in contact with the affected persons (one school setting) played major parts in the low transmission rate reported. In all the affected schools, self‐isolation at home was not recommended or adopted. Other preventive strategies that helped to contain mpox spread include vaccination of high‐risk students. Ladhani et al.^26^ indicated that not all suspected cases categorized as high risk who were offered (186 students) the Modified Vaccinia Ankara‐Bavarian Nordic (MVA‐BN) vaccine received it. A total of 21 students (Primary school—10; Nursery school—7; Reception year—4) with ages ranging from 2 to 11 years received a single dose of the vaccine. The vaccine was administered not less than 7 days and not later than 14 days after having contact with the positive case. The rate of vaccine uptake was 11%.

### 3.2 Evidence/research gaps

Only one report on the national monkeypox outbreak in England satisfied the selection criteria and was assessed in this review. Although, the eligible publication is a nonintervention study as it reported epidemiological and surveillance information. It described how suspected cases of mpox infection were managed in some school settings. This review indicates a major gap in literature specifically, the research gap on school‐based interventions on mpox infection. Results from this scoping review did not reveal whether any intervention programs on mpox were introduced in the affected schools and whether such intervention had significant effects on low mpox transmission among young children.^26^ While the authors reported low vaccine uptake, reasons for the low uptake were not reported and no study focused on this aspect of mpox control was identified in our review. Apart from isolation, Ladhani et al. did not report other preventive mechanisms adopted in the study population.

Furthermore, given the high risks that young school students are exposed to daily, there is no scientific literature detailing any educational interventions or programs targeting behavioral change communication. We are not aware of any scientific communication on the effectiveness or otherwise of such programs. Research on school programs targeted at modifying/improving knowledge, awareness and practices among students are also lacking. Largely, interventions on health behavior or student‐centered interventions are undocumented.

Moreover, no study reported hand hygiene and cleanliness interventions, which are essential to infection prevention as observed during the COVID‐19 outbreak. Studies on Africa where most of the endemic countries are located are largely absent.

## 4 DISCUSSION

Mpox virus is traditionally endemic in Western and Central African regions. Its human transmission was not identified in other parts of the world until 2022 when mpox cases were confirmed in non‐endemic nations spreading across Europe, North America and parts of the Middle‐East.^27^, ^28^ Unlike previous outbreaks, the 2022 mpox outbreak was not connected to travel and imported animals.^29^ Close contacts such as through sexual encounters and contact with respiratory droplets remain the major risk factor for infection transmission.^30^

Sexual encounters, and in particular, sexual orientation have been implicated in mpox transmission.^31^, ^32^ Islam et al.^31^ maintained that sexual minorities (especially LGBTQ [lesbian, gay, bisexual, transgender, and queer (or questioning)] and MSM [men who have sex with men] communities) are disproportionately affected by mpox infection. Islam et al.^31^ pointed to a possible association between epidemiology of mpox infection and sexual orientation patients. Low et al.^32^ found similar findings about the disproportionately high burden of mpox among sexual minorities in a multicountry outbreak. The study documented that mpox transmission occurs through sexual contact among sexual minorities (both penetrative and nonpenetrative), more than casual skin‐to‐skin contact.^32^

The study reviewed here reported epidemiology and mpox outbreak management strategies and infection prevention and control efforts in some school settings in England subsequent to four different cases of exposure. Following the 28‐day follow‐up window which involved isolation and separation of no less than 440 exposed persons, zero case of mpox infection was identified as none of the low, medium, and high‐risk groups were infected. This indicates a low transmission rate in school/student population. Although the finding of our scoping review should be taken with caution due to limited scientific evidence on the topic. However, the epidemiology of mpox infection in England reflects a similar trend as almost all the cases occurred in adult population. Out of the 3390 infected persons, 99% and 1% were men and women, respectively. Whereas just one minor (0−15 years) was confirmed positive.^26^

Studies have linked human transmission of the mpox virus to prolonged exposure and close physical contact with positive cases.^29^ Whereas our review shows that the suspected cases only had fleeting contact with the positive cases in the four school settings, hence the low transmission risks. Nevertheless, the risks of prolonged interactions in school settings often raise concerns among public health experts as the school environment may escalate community transmission of infections.^33^ Experiences during COVID‐19 outbreak show that indoor settings such as schools are “potential infection hotspots” due to the immense close contacts, learning and leisure activities that characterize them. Moreover, school children are also major vehicles of infection transmission.^33^, ^34^ Despite the low risk of mpox infection among school students, schools often serve as a pool of viral infection pathogens.

Moreover, through close contact, children can likewise transmit infections to adults, hence the importance of school‐based intervention for the control of infectious diseases in endemic countries. Probable community transmission of infectious diseases through the school settings is always a public health concern because school children and young adults up to the secondary school level constitute a significant percentage of the population.^35^ Notwithstanding the low risk of mpox infection among children and young adults, in the USA, some mpox infections have been confirmed among children (0−15 years: 57) and young adults (16−20 years: 605) who had eczema and were immunosuppressed.^30^

Similarly, adults who are often in direct contact with children such as school administrators, teachers, and parents are more susceptible to mpox infection. Even children who are immunocompromised and prone to certain skin conditions are at high risk of infection and are likely to develop complications when exposed to the virus.^30^ It is also important that Africa health authorities take school‐based intervention seriously to reduce the burden of mpox on school‐aged children. Therefore, school‐based interventions are important for preventing infection spread and promoting the health of parents, teachers, and others in society.^35^

### 4.1 Limitations and strengths of this review

The most fundamental limitation of the review is a lack of a pool of evidence due to limited research on school‐based intervention on mpox. For infectious diseases, physical contact is a major transmission route, hence, the school environment could constitute a cesspool of risk transmission. Therefore, the major strength of this review is that it is a global scoping review in the wake of the global mpox emergency. Moreso, the review will draw the attention of relevant stakeholders to the need for school‐based interventions as a fundamental mpox control strategy in promoting the health of pupils, parents, teachers, and others.

## 5 CONCLUSION

Mpox is a global health emergency that has spread to up to 110 countries. This is the first time it has spread globally beyond West and Central African countries. Unlike previous outbreaks, the 2022 mpox outbreak comes with a new spread dimension—through close sexual encounters, especially among sexual minorities (such as LGBT and MSM). The implication is that mpox is sexually transmissible, which should attract significant focus in global health intervention. The review suggests low human‐to‐human transmission in school settings. This review shows that school‐based interventions, involving isolation of exposed population, are highly effective in curtailing the spread of mpox within the school setting. While the inference is basd on limited evidence, it is, however, a fact that the school setting could also serve as a point of disseminating information and propagating vaccination programs, among other interventions. Hence, there is a need for empirical studies in school settings concerning mpox transmissions and interventions. The mpox vaccine program should, significantly, consider at‐risk populations including children and sexual minorities. With the call for a multisectoral approach in the fight against mpox, intervention decision‐makers can leverage the potential of school settings in propagating health information to ensuing preventive actions. Therefore, school‐based interventions are crucial in promoting the health of pupils, parents, teachers, and others.

## AUTHOR CONTRIBUTIONS

**Jimoh Amzat**: Conceptualization; formal analysis; investigation; project administration; resources; supervision; validation; visualization; writing—original draft; writing—review and editing. **Kehinde K. Kanmodi**: Conceptualization; data curation; funding acquisition; investigation; methodology; project administration; resources; software; supervision; validation; visualization; writing—original draft; writing—review and editing. **Kafayat Aminu**: Data curation; formal analysis; investigation; resources; validation; visualization; writing—original draft. **Eyinade A. Egbedina**: Data curation; investigation; resources; software; validation.

## CONFLICTS OF INTEREST STATEMENT

Kehinde K. Kanmodi is an Editorial Board member of Health Science Reports and a coauthor of this article. To minimize bias, he was excluded from all editorial decision‐making related to the acceptance of this article for publication. The authors declare no conflict of interest.

## ETHICS STATEMENT

This study did not collect data from human or animal subjects but an open research repository.

## TRANSPARENCY STATEMENT

The corresponding author, Kehinde K. Kanmodi, affirms that this manuscript is an honest, accurate, and transparent account of the study being reported; that no important aspects of the study have been omitted; and that any discrepancies from the study as planned (and, if relevant, registered) have been explained.

## Data Availability

Data sharing is not applicable to this article as no new data were created or analyzed in this study.

## 1

**Table A1:** Search string for PubMed database search.

Tag	Subject search	Search string
#1	Monkeypox	(Monkeypox [Title/Abstract]) OR (Mpox [Title/Abstract]) OR (Vírus da Varíola dos Macacos [Title/Abstract]) OR (Poxvirus do Macaco [Title/Abstract]) OR (Vírus da Varíola dos Símios [Title/Abstract]) OR (Vírus de Monkeypox [Title/Abstract]) OR (pox [Title/Abstract]) OR (orthopoxvirus [Title/Abstract])
#2	School	(Secondary school [Title/Abstract]) OR (high school [Title/Abstract]) OR (middle school [Title/Abstract]) OR (elementary school [Title/Abstract]) OR (primary school [Title/Abstract]) OR (teacher [Title/Abstract]) OR (student [Title/Abstract]) OR (pupil [Title/Abstract]) OR (classroom [Title/Abstract])
#3	#1 AND #2	(#1) AND (#2)

**Table A2:** Search string for SCOPUS database search.

Tag	Subject search	Search string
#1	Monkeypox	(TITLE‐ABS‐KEY [“monkeypox”] OR TITLE‐ABS‐KEY [mpox] OR TITLE‐ABS‐KEY [“Vírus da Varíola dos Macacos”] OR TITLE‐ABS‐KEY [“Poxvirus do Macaco”] OR TITLE‐ABS‐KEY [“Vírus da Varíola dos Símios”] OR TITLE‐ABS‐KEY [“Vírus de Monkeypox”] OR TITLE‐ABS‐KEY [pox] OR TITLE‐ABS‐KEY [orthopoxvirus])
#2	School	(TITLE‐ABS‐KEY [“secondary school”] OR TITLE‐ABS‐KEY [“high school”] OR TITLE‐ABS‐KEY [“middle school”] OR TITLE‐ABS‐KEY [“elementary school”] OR TITLE‐ABS‐KEY [“primary school”] OR TITLE‐ABS‐KEY [teacher] OR TITLE‐ABS‐KEY [student] OR TITLE‐ABS‐KEY [pupil] OR TITLE‐ABS‐KEY [classroom] OR TITLE‐ABS‐KEY [school] OR TITLE‐ABS‐KEY [nursery] OR TITLE‐ABS‐KEY [kindergarten])
#3	#1 AND #2	(TITLE‐ABS‐KEY [“monkeypox”] OR TITLE‐ABS‐KEY [mpox] OR TITLE‐ABS‐KEY [“Vírus da Varíola dos Macacos”] OR TITLE‐ABS‐KEY [“Poxvirus do Macaco”] OR TITLE‐ABS‐KEY [“Vírus da Varíola dos Símios”] OR TITLE‐ABS‐KEY [“Vírus de Monkeypox”] OR TITLE‐ABS‐KEY [pox] OR TITLE‐ABS‐KEY [orthopoxvirus]) AND (TITLE‐ABS‐KEY [“secondary school”] OR TITLE‐ABS‐KEY [“high school”] OR TITLE‐ABS‐KEY [“middle school”] OR TITLE‐ABS‐KEY [“elementary school”] OR TITLE‐ABS‐KEY [“primary school”] OR TITLE‐ABS‐KEY [teacher] OR TITLE‐ABS‐KEY [student] OR TITLE‐ABS‐KEY [pupil] OR TITLE‐ABS‐KEY [classroom] OR TITLE‐ABS‐KEY [school] OR TITLE‐ABS‐KEY [nursery] OR TITLE‐ABS‐KEY [kindergarten])

**Table A3:** Search string for other database (AMED**—**the allied and complementary medicine database; child development and adolescent studies; dentistry and oral sciences source; SPORTDiscus with Full Text; APA PsycArticles; psychology and behavioral sciences collection; APA PsycInfo and CINAHL Ultimate) search via EBSCO interface.

Tag	Subject search	Search string
S1	Monkeypox	AB Monkeypox OR AB Mpox OR AB Vírus da Varíola dos Macacos OR AB Poxvirus do Macaco OR AB Vírus da Varíola dos Símios OR AB Vírus de Monkeypox OR AB pox OR AB orthopoxvirus
S2	School	AB Secondary school OR AB high school OR AB middle school OR AB elementary school OR AB primary school OR AB teacher OR AB student OR AB pupil
S3	S1 AND S2	(S1) AND (#S2)
